# Supplementary figures and images for: Antibody responses induced by SHIV infection are more focused than those induced by soluble native HIV-1 envelope trimers in non-human primates
Source: PLoS Pathog. 2021 Aug 25;17(8):e1009736. doi: 10.1371/journal.ppat.1009736 (PMC8423243; doi:10.1371/journal.ppat.1009736)

## Immunized NHP ROp15

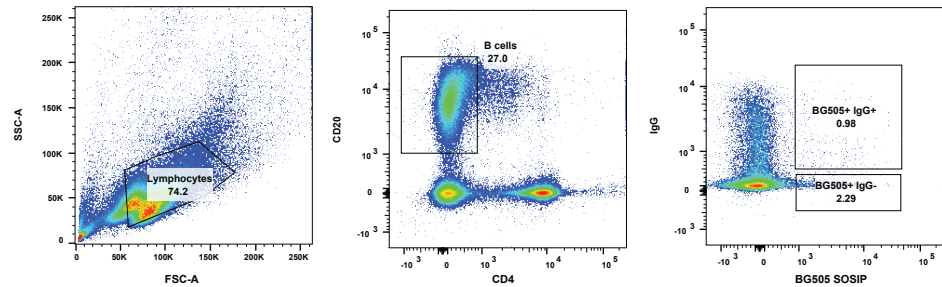

## Sorted cells

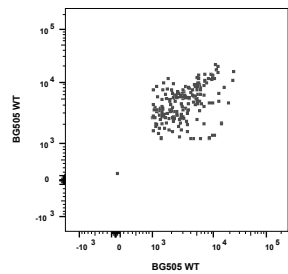

## Infected NHP 6454

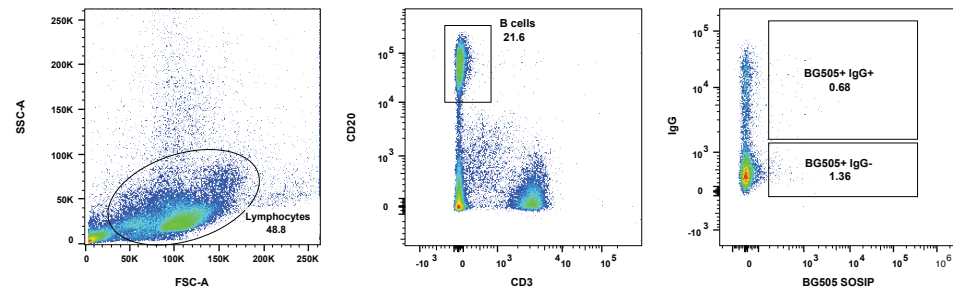

## Sorted cells

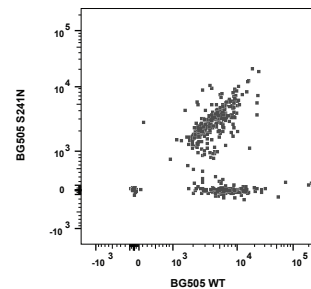

Supplement: S1 Fig — For the immunized NHPs, BG505-specific B cells were selected by fluorescence activated cell sorting using two differently labeled BG505 SOSIPs. The gating strategy of NHP ROp15 is shown here (left). In contrast, BG505-specific B cells from the BG505 SHIV-infected NHPs were sorted with a BG505 SOSIP and BG505 SOSIP S241N trimer. The gating strategy of NHP 6454 is shown here (right). (PDF) [file ppat.1009736.s001.pdf]

A

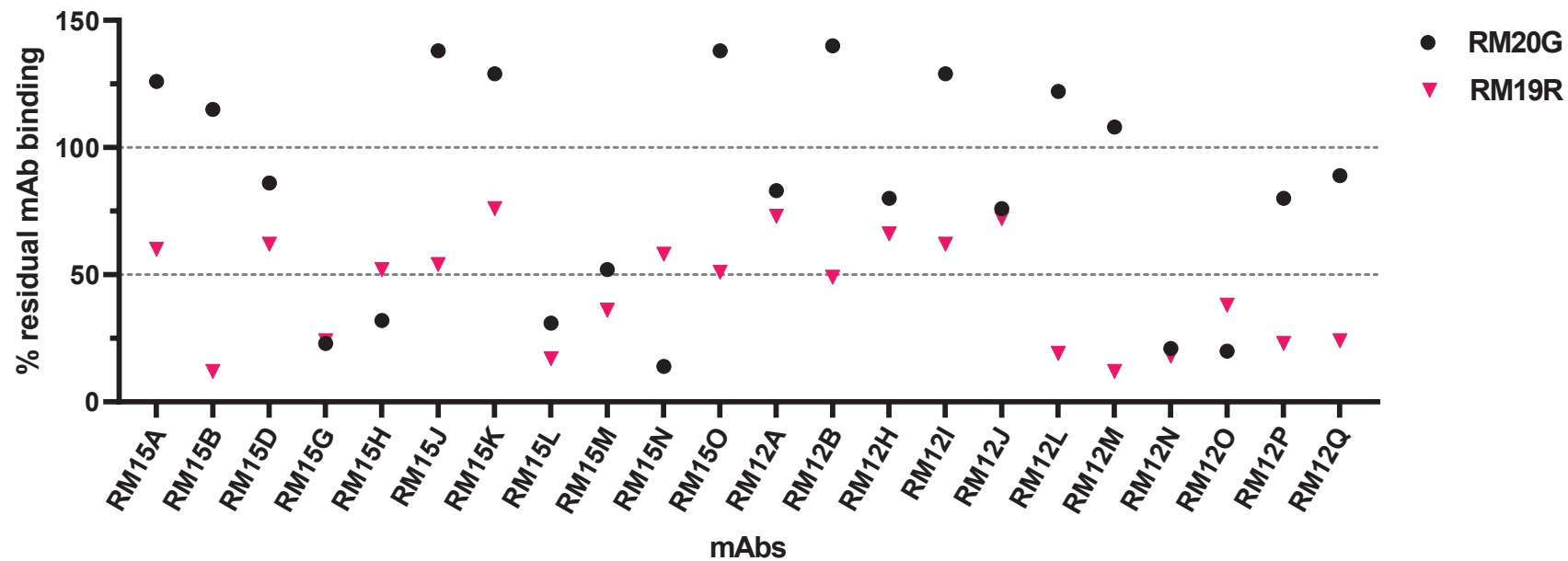

B

RM15A

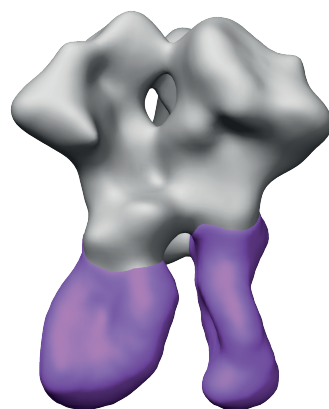

Side view

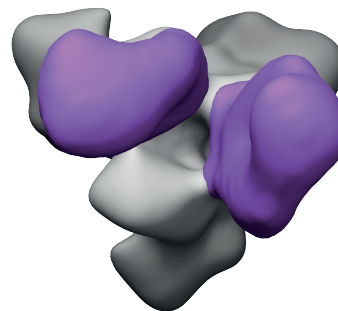

Bottom view

Supplement: S3 Fig — (A) Competitive ELISA with base-targeting mAbs RM20G and RM19R isolated from BG505 SOSIP.664 immunized NHPs in a previous study. (B) Negative-stain electron microscopy 3D reconstruction of RM15A (purple) in complex with BG505 SOSIP (grey). RM15A was isolated from NHP ROp15. (PDF) [file ppat.1009736.s003.pdf]
