# Supplementary material for: Antibody responses induced by SHIV infection are more focused than those induced by soluble native HIV-1 envelope trimers in non-human primates
Source: PLoS Pathog. 2021 Aug 25;17(8):e1009736. doi: 10.1371/journal.ppat.1009736 (PMC8423243; doi:10.1371/journal.ppat.1009736)
Supplement: S2 Fig — To map the epitopes of the NAbs, we performed neutralization assays with a variety of virus variants. For each mAb, the IC50 value against each pseudovirus variant relative to the BG505.T332N parental virus is given. This value is indicated as the relative inhibitory concentration 50 (RIC50). If the NAbs were unable to neutralize a virus variant, the value indicates the IC50 divided by the highest concentration that was used in the assay that gave no neutralization. (PDF) [file ppat.1009736.s002.pdf]

Immunization

Animal ID	Antibody ID	IC$_{50}$ BG505	Relative IC$_{50}$ N611A	S241N	P291T	S241N P291T	T465N	133aN
ROp15	RM15C	1.4	0.52	1.4	0.57	0.87	1.3	>35
	RM15F	0.20	0.95	1.4	0.90	1.2	0.60	53
	RM15E	0.12	4.2	2.9	27	19	368	1.7
	RM15I	21	0.065	>2.4	>2.4	>2.4	>2.4	>2.4
99-12	RM12K	3.1	1.1	1.6	3.2	2.2	11	0.61
	RM12F	>50	0.013	1.0	1.0	1.0	1.0	1.0

RIC$_{50}$	
	<0.33
	≥ 0.33 ≤3.0
	>3.0 ≤ 20
	>20
	No neutralization

Infection

Animal ID	Antibody ID	IC$_{50}$ BG505	Relative IC$_{50}$ N611A	S241N	P291T	S241N P291T	T465N	133aN
6454	RM54B1	0.016	1.0	2.0	2.5	2.5	2.5	>3125
	RM54B2	0.022	1.0	2.0	1.5	3.5	1.0	>2272
	RM54B3	0.027	1.0	4.0	4.7	3.3	3.3	>1851
	RM54B4	0.039	0.25	3.0	4.8	4.0	2.3	>1282
	RM54B5	0.083	0.50	2.3	2.0	2.0	1.3	>602
	RM54B6	0.026	1.0	3.0	1.0	1.3	1.7	>1923
	RM54B7	0.031	1.7	4.7	2.0	2.0	2.3	>1612
	RM54B8	0.053	0.80	4.8	11	3.0	1.2	>943
	RM54B9	0.21	0.62	0.76	1.7	0.10	0.10	>238
	RM54B10	0.016	1.1	4.0	1.0	0.50	1.2	>3125
	RM54B11	0.054	0.42	1.2	0.80	1.6	0.80	>929
	RM54B12	0.15	0.54	2.1	0.80	0.87	0.73	>333
	RM54B13	0.030	0.33	2.0	1.3	1.0	1.0	>1667
	RM54B14	0.043	0.86	4.3	4.0	4.0	2.5	>1162
	RM54B15	0.10	0.78	3.9	4.0	2.2	2.7	>500
	RM54B16	0.010	0.33	0.86	0.75	0.67	0.75	>5000
	RM54B17	0.027	0.69	1.7	2.0	2.4	1.2	>1851
	RM54B18	0.011	1.0	9.0	82.0	5.0	10.0	>4545
	RM54B19	0.33	0.85	1.4	5.1	2.9	>152	>152
	RM54B20	11	1.3	2.6	>5.0	>5.0	1.9	>4.6
	RM54B21	12	0.85	>4.0	>4.0	1.8	0.72	>4.1
	RM54B22	12	1.1	1.9	>4.0	>4.0	2.5	>4.1
	RM54B23	7.1	0.06	4.4	7.0	1.1	5.0	>7.0
43335	RM35A1	0.034	0.67	1.3	1.7	1.3	1.0	>1470
	RM35A2	0.070	0.14	0.57	0.29	0.71	0.57	>714
6446	RM46B1	17	0.63	2.8	>2.9	>2.9	>2.9	>2.9
	RM46B2	26	0.63	>1.9	>1.9	>1.9	>1.9	>1.9
