# Supplementary material for: Antibody responses induced by SHIV infection are more focused than those induced by soluble native HIV-1 envelope trimers in non-human primates
Source: PLoS Pathog. 2021 Aug 25;17(8):e1009736. doi: 10.1371/journal.ppat.1009736 (PMC8423243; doi:10.1371/journal.ppat.1009736)
Supplement: S1 Table — (PDF) [file ppat.1009736.s005.pdf]

Antibody ID	Animal ID	LC type	Epitope	VH gene	DH gene	JH gene	CDRH3 (aa)	CDRH3 length	V identity (%)	SHM VH (%)	VL gene	JL gene	CDRL3 (aa)	CDRL3 length	VL identity (%)	SHM VL (%)
RM15A	R0p15	VK	Base	IGHV1-AAU*01_S5608	IGHD2-35*01,IGHD2-8*01,IGHD2-I2	IGHJ4*01	ARSSEWDQLPGGQLVVSGFDH	21	94,2	5,8	IGKV1-ADF*01_S1146	IGKJ4-I4	QQHNTYPLT	9	93,0	7,0
RM15B	R0p15	VK	Base	IGHV3-AAB*01_S6942	IGHD6-29*01	IGHJ5-2*01	IRARKISATGPLGNSLDV	18	95,6	4,4	IGKV2-AEM*01	IGKJ3-I3	MQGTQVPFT	9	97,7	2,3
RM15C	R0p15	VK	V1 region	IGHV3-ADO*01,LJI.Rh_IGHV3.88.a	IGHD1-7*01,LJI.Rh_IGHD1.5,LJI.Rh_IGHD1.6	IGHJ5-1*01	TRERYASWSPYNWIDV	16	93,7	6,3	IGKV1-ABZ*01,IGKV1-ACI*01,IGKV1-AEG*01	IGKJ1-I1	QQCYSAPPT	9	93,0	7,0
RM15D	R0p15	VK	Base	IGHV4-ABB*01_S8200	LJI.Rh_IGHD3.41	IGHJ2-I1	ARGKRIWTFYYHYCYFDV	18	92,9	7,1	LJI.Rh_IGKV1.63.a	IGKJ4-I4	QHGYDILALTF	11	97,2	2,8
RM15E	R0p15	VK	C3/V5	IGHV4-AFB*01	IGHD2-30*01,IGHD2-I5,IGHD6-34*01	IGHJ4*01	ARGPLGS	7	93,6	6,4	IGKV1-AAY*01	IGKJ3-I3	RRFCAVVA	8	94,7	5,3
RM15F	R0p15	VK	gp41/gp120 interface	IGHV4-AFQ*01	IGHD1-1*01,IGHD4-27*02,IGHD5-10*01	IGHJ2-I1	AKHPSGGPAYWYFDL	15	91,0	9,0	IGKV1-S57	IGKJ2-I2	HHSYGSPYSF	10	93,0	7,0
RM15G	R0p15	VK	Base	IGHV4-AGU*01	IGHD4-4*02	IGHJ4*01	ARLRSFATSYGDYFDY	16	89,9	10,1	IGLV2-ABJ*01_S9052	IGLJ1-S1	CSYAGSNTYI	10	97,3	2,7
RM15H	R0p15	VL	Base	IGHV4-ABB*01_S8200	IGHD1-19*01,IGHD1-19*02	IGHJ3*01	ARNWCAATAGTCAFDF	16	92,9	7,1	LJI.Rh_IGLV2.29	IGLJ3*01	SSYAGSHTWV	10	97,7	2,3
RM15I	R0p15	VL	241	IGHV4-AEB*01	IGHD3-3*01	IGHJ4*01	AREGTSHNFWSGFPEGRYFDY	21	91,7	8,3	IGLV2-ABU*01,LJI.Rh_IGLV2.12	IGLJ1-S1	NSYGTSRTYI	10	95,9	4,1
RM15J	R0p15	VL	Base	IGHV4-AEX*01	IGHD4-15*01,IGHD4-22*01,IGHD4-32*01	IGHJ5-1*01	ASLQDLTTPYNRFNV	15	92,5	7,5	LJI.Rh_IGLV2.112	IGLJ1*01,IGLJ1-S1	CSYTIFSAFIF	11	94,9	5,1
RM15K	R0p15	VL	Unknown	IGHV4-AEX*01_S8720	IGHD3-9*01,LJI.Rh_IGHD3.15	IGHJ4*01	ARMYEDDYGFYNTVRNGWGFDS	22	92,2	7,8	IGLV1-ACN*02	IGLJ3-S3	QTYDSTLSTHVL	12	96,3	3,7
RM15L	R0p15	VL	Base	IGHV4-AFQ*01	IGHD2-13*01,IGHD2-I3	IGHJ4*01	VRHPGALTAPDMVN	14	93,0	7,0	IGLV11-AAY*02	IGLJ3-S3	QVYGGGASLF	10	96,1	3,9
RM15M	R0p15	VL	Base	IGHV4-AGR*01_S4099	IGHD6-6*01	IGHJ4*01	ARERVSSAWYAVGYYFDF	18	93,0	7,0	IGLV2-ABE*01_S2946	IGLJ1*01,IGLJ1-S1	SSYAGSNTFIF	11	96,6	3,4
RM15N	R0p15	VL	Base	IGHV4-AGU*01	LJI.Rh_IGHD1.6	IGHJ3*01	ARLIRGSTSWNDAFDF	16	89,2	10,8	IGLV2-ABJ*01_S9052	IGLJ1-S1	SSLAGTNTYV	10	93,9	6,1
RM15O	R0p15	VL	Base	IGHV5-AER*01,LJI.Rh_IGHV5.157	IGHD3-3*01	IGHJ2-I1	ARMAYYNFWSGYYTSRDWYFDL	22	96,9	3,1	IGLV5-AAX*01	IGLJ1-S1	MIWHNNAYI	9	99,7	0,3
RM12A	99-12	VK	Base	IGHV1-AAU*02,IGHV1-AAU*02_S4606	IGHD4-27*01,LJI.Rh_IGHD4.24	IGHJ6*01	AREGQLQYGYALD	13	92,2	7,8	LJI.Rh_IGKV1.49	IGKJ2-I2	QQRHSHPHS	9	95,4	4,6
RM12B	99-12	VK	Base	IGHV3-ADL*01	IGHD2-25*01,IGHD2-I4	IGHJ6*01	ARGPVLVFTAMLN	13	93,9	6,1	IGKV2-AEB*01_S5603	IGKJ4-I4	MQALEFPLT	9	95,7	4,3
RM12C	99-12	VK	289	IGHV4-ADD*01_S5847	LJI.Rh_IGHD6.35	IGHJ4*01	ARWQTPFDY	9	87,4	12,6	IGKV1-AAL*01_S2543	IGKJ4-I4	QQHNSYPLT	9	96,9	3,1
RM12D	99-12	VK	289	IGHV4-ADG*01	IGHD1-19*01,IGHD1-19*02	IGHJ4*01	ARKQSNFDS	9	88,9	11,1	IGKV1-AAL*01_S2543	IGKJ4-I4	QQHNTYTLT	9	96,8	3,2
RM12E	99-12	VK	289	IGHV4-ADG*01	IGHD6-24*01	IGHJ4*01	VRRQSNFDP	9	88,9	11,1	IGKV1-AAY*01	IGKJ1-I1	QQHNGDPPT	9	95,8	4,2
RM12F	99-12	VK	N611/FP	IGHV4-AEX*01_S9508	IGHD3-Kc,LJI.Rh_IGHD3.16	IGHJ5-1*01	ARDVYYYSGLRSPNWFDV	18	91,6	8,4	IGKV1-S57	IGKJ3-I3	QHSYGTPYT	9	95,8	4,2
RM12G	99-12	VK	Base	IGHV4-AGR*01_S5203	IGHD3-9*01,IGHD6-34*01,LJI.Rh_IGHD3.15	IGHJ4*01	VRRGGRAFSGGVYFDY	16	90,3	9,7	LJI.Rh_IGKV1.26	IGKJ4-I4	QQGNSHPLT	9	95,8	4,2
RM12H	99-12	VK	Base	IGHV4-S11_S0312	IGHD3-14*01,IGHD3-Kc,LJI.Rh_IGHD3.16	IGHJ6*01	ARDVRWGGLTASYYSDYGLD	20	95,0	5,0	IGKV1-ADO*01_S6906	IGKJ3-I3	LQGYDTPFT	9	95,8	4,2
RM12I	99-12	VK	Unknown	LJI.Rh_IGHV2.69	IGHD5-23*01,IGHD6-29*01,IGHD6-6*01	IGHJ4*01	ARVLAAGGDQ	10	92,7	7,3	IGKV2-AEB*01_S5603	IGKJ1-I1	MQTLEFPWT	9	99,0	1,0
RM12J	99-12	VK	Base	LJI.Rh_IGHV3.88.a_S9033	IGHD3-14*01,IGHD3-Kc,LJI.Rh_IGHD3.16	IGHJ4*01	AVINSRLSV	9	92,2	7,8	IGKV2-ADV*01	IGKJ2-I2	MQALQTPYS	9	97,3	2,7
RM12K	99-12	VK	gp41/gp120 interface	LJI.Rh_IGHV4.79.a_S9501	IGHD3-3*01	IGHJ4*01	ARDPRRLAIFGVVIYFDY	18	91,2	8,8	IGKV6-ABK*01	IGKJ4-I4	QQSSSFPLN	9	98,3	1,7
RM12L	99-12	VL	Base	IGHV3-ADL*01	IGHD2-13*01,IGHD2-25*01,IGHD2-I3	IGHJ4*01	VRQPPVLASSDPKYYFDF	18	92,1	7,9	IGLV2-ABE*01_S2946	IGLJ3*01	SSYAGRNAYWV	11	94,2	5,8
RM12M	99-12	VL	Base	IGHV4-AEX*01	IGHD2-13*01,IGHD2-I3	IGHJ4*01	ARLRDIYGTYVTY	13	92,9	7,1	IGLV11-AAY*01	IGLJ2-S2,IGLJ3-S3	QVYDSSANVI	10	92,6	7,4
RM12N	99-12	VL	Base	IGHV4-AGR*01_S4099	IGHD6-34*01	IGHJ6*01	ARDQSGAWSDYYGLD	15	94,9	5,1	IGLV11-AAY*01	IGLJ6-S6	RVYDSSGDV	9	94,8	5,2
RM12O	99-12	VL	Base	IGHV4-AGU*01_S6650	IGHD6-34*01	IGHJ4*01	ARHKVGGAWSMPQHYFEY	18	90,9	9,1	IGLV2-ABE*01_S2946	IGLJ1*01,IGLJ1-S1	TSYAGSNSFIF	11	94,9	5,1
RM12P	99-12	VL	Base	IGHV4-Korf4_S3924	IGHD3-3*01	IGHJ5-1*01	VRRGITIFGGSNDWFDV	17	93,3	6,7	IGLV11-AAY*02	IGLJ1*01,IGLJ1-S1	QVHDSDANII	10	94,9	5,1
RM12Q	99-12	VL	Base	IGHV4-S11_S0312	LJI.Rh_IGHD4.21	IGHJ6*01	AKLNSITSHYHGLHPWGQ	18	94,9	5,1	IGLV11-AAY*01	IGLJ6-S6	QIYDRSANIV	10	95,2	4,8
RM54A	6454	VK	289	IGHV4-AGU*01_S6650	IGHD6-34*01	IGHJ4*01	ARWAAADFDF	10	90,8	9,2	IGKV1-AAL*01	IGKJ2-I2	QHHYRYPYS	9	94,0	6,0
RM54B1	6454	VL	V1 region	IGHV3-AAB*02	IGHD3-26*01,LJI.Rh_IGHD3.18	IGHJ6*01	TRERGCGGGACHNYYGSGYHYDGLE	25	94,9	5,1	IGLV10-AAO*01	IGLJ3*01	SAWDTSLSAWV	11	96,6	3,4
RM54B2	6454	VL	V1 region	IGHV3-AAB*02	IGHD3-26*01,LJI.Rh_IGHD3.18	IGHJ6*01	SREGGCGSGVCHDWYGSGKYYIGLD	25	96,3	3,7	IGLV10-AAO*01	IGLJ3*01	SAWDTSLSAWV	11	92,2	7,8
RM54B3	6454	VL	V1 region	IGHV3-AAB*02	IGHD3-26*01,LJI.Rh_IGHD3.18	IGHJ6*01	TRERGCGGGACHNYYGSGYYYDGLE	25	95,9	4,1	IGLV10-AAO*01	IGLJ3*01	SAWDTSLSVWV	11	96,2	3,8
RM54B4	6454	VL	V1 region	IGHV3-AAB*02_S9425	IGHD2-35*01,IGHD2-8*01,IGHD2-I2	IGHJ6*01	ARERGCSGGVCQNWYASGYYYQALD	25	92,2	7,8	IGLV10-AAO*01	IGLJ3*01	SAWDTVLTAWL	11	93,9	6,1
RM54B5	6454	VL	V1 region	IGHV3-AAB*02	IGHD3-26*01,LJI.Rh_IGHD3.18	IGHJ6*01	TRERGCGGGACHNYYGSGYHYDGLE	25	95,3	4,7	IGLV10-AAO*01	IGLJ3*01	SAWDTSLSAWV	11	96,6	3,4
RM54B6	6454	VL	V1 region	IGHV3-AAB*02	IGHD2-35*01,IGHD2-8*01,IGHD2-8*02	IGHJ6*01	SREGGCGSGVCYYWYGSGKYCIGLD	25	95,9	4,1	IGLV10-AAO*01	IGLJ3*01	SAWDTGLGAWV	11	93,2	6,8
RM54B7	6454	VL	V1 region	IGHV3-AAB*02	IGHD2-8*01,IGHD2-8*02,IGHD2-I2	IGHJ6*01	SREGGCGSGVCYDWYGSGKYYNGLD	25	95,3	4,7	IGLV10-AAO*01	IGLJ3*01	SAWDTSLSAWV	11	93,9	6,1
RM54B8	6454	VL	V1 region	IGHV3-AAB*02	IGHD2-35*01,IGHD2-8*01,IGHD2-I2	IGHJ6*01	TREGGCSGGVCNNYYGSGYHYHGLD	25	95,9	4,1	IGLV10-AAO*01	IGLJ3*01	SAWDTSLSAWV	11	95,6	4,4
RM54B9	6454	VL	V1 region	IGHV3-AAB*02	IGHD3-26*01,LJI.Rh_IGHD3.18	IGHJ6*01	TREGGCSGGVCNDYYGSGYHYHGLD	25	94,6	5,4	IGLV10-AAO*01	IGLJ3*01	SAWDTSLSAWV	11	96,3	3,7
RM54B10	6454	VL	V1 region	IGHV3-AAB*02	IGHD3-26*01,LJI.Rh_IGHD3.18	IGHJ6*01	TRERGCGGGACHDYYGSGYHYDGLE	25	94,9	5,1	IGLV10-AAO*01	IGLJ3*01	SAWDTSLSAWV	11	96,3	3,7
RM54B11	6454	VL	V1 region	IGHV3-AAB*02	IGHD2-8*01,IGHD2-8*02,IGHD2-I2	IGHJ6*01	SREGGCGSGVCYDWYGSGKYYNGLD	25	97,0	3,0	IGLV10-AAO*01	IGLJ3*01	SAWDTGLSAWV	11	93,2	6,8
RM54B12	6454	VL	V1 region	IGHV3-AAB*02	IGHD2-35*01,IGHD2-I6	IGHJ6*01	SREGGCSGGVCYNYYGSGYHYHGLD	25	94,9	5,1	IGLV10-AAO*01	IGLJ3*01	SAWDTSLSAWV	11	93,5	6,5
RM54B13	6454	VL	V1 region	IGHV3-AAB*02,IGHV3-AAB*02_S9425	IGHD2-35*01,IGHD2-8*01,IGHD2-I2	IGHJ6*01	TRERGCGGGACHNYYGSGYYYDGLE	25	95,9	4,1	IGLV10-AAO*01	IGLJ3*01	SAWDTSLSAWV	11	97,3	2,7
RM54B14	6454	VL	V1 region	IGHV3-AAB*02	IGHD2-35*01,IGHD2-8*01,IGHD2-I2	IGHJ6*01	TREGGCSGGVCNDYYGSGYHYHGLD	25	95,9	4,1	IGLV10-AAO*01	IGLJ3*01	SAWVTGLSAWV	11	94,6	5,4
RM54B15	6454	VL	V1 region	IGHV3-AAB*02	IGHD2-8*01,IGHD2-I2	IGHJ6*01	SREGGCSGGVCYDYYGSGYHYQGLD	25	95,9	4,1	IGLV10-AAO*01	IGLJ3*01	SAWDTNLSAWV	11	93,2	6,8
RM54B16	6454	VL	V1 region	IGHV3-AAB*02	IGHD2-8*01,IGHD2-I2	IGHJ6*01	ARERGCSGGACYDWYFSGYHYQGLD	25	93,9	6,1	IGLV10-AAO*01	IGLJ3*01	SAWDTGLSAWL	11	95,2	4,8
RM54B17	6454	VL	V1 region	IGHV3-AAB*02	IGHD3-26*01,LJI.Rh_IGHD3.18	IGHJ6*01	TRERGCGGGACHNYYGSGYYYDGLE	25	95,9	4,1	IGLV10-AAO*01	IGLJ3*01	SAWDTSLSAWV	11	97,3	2,7
RM54B18	6454	VL	V1 region	IGHV3-AAB*02	IGHD2-35*01,IGHD2-8*01,IGHD2-I2	IGHJ6*01	SREGGCSGGVCHNYYGSGYHYQGLD	25	95,3	4,7	IGLV10-AAO*01	IGLJ3*01	SAWDTSLSAWV	11	93,2	6,8
RM54B19	6454	VL	V1 region	IGHV3-AAB*02	IGHD2-35*01,IGHD2-I6	IGHJ6*01	SREGGCSGGVCYNYYGSGYHYQGLD	25	95,6	4,4	IGLV10-AAO*01	IGLJ3*01	SAWDTNLSAWV	11	93,5	6,5
RM54B20	6454	VL	V1 region	IGHV3-AAB*02	IGHD2-35*01,IGHD2-8*01,IGHD2-I2	IGHJ6*01	TREGGCSGGVCQNWYYSGRHFHGLD	25	95,3	4,7	IGLV10-AAO*01	IGLJ3*01	SSWDTTLSAWV	11	94,9	5,1
RM54B21	6454	VL	V1 region	IGHV3-AAB*02	IGHD2-8*01,IGHD2-I2	IGHJ6*01	SREGGCSGGVCYDYYGSGYHYQGLD	25	96,3	3,7	IGLV10-AAO*01	IGLJ3*01	SAWDTGLSAWV	11	93,2	6,8
RM54B22	6454	VL	V1 region	IGHV3-AAB*02	IGHD2-35*01,IGHD2-8*01,IGHD2-I2	IGHJ6*01	VREGGCSGGVCDNWYYSGSRYHGLD	25	94,9	5,1	IGLV10-AAO*01	IGLJ3*01	SGWDTGLGAWL	11	93,5	6,5
RM54B23	6454	VL	V1 region	IGHV3-AAB*02,IGHV3-AAB*02_S9425	IGHD2-35*01,IGHD2-I6	IGHJ6*01	TREKGRGRDCSSGVCYNYYGSGYYYQALD	29	94,6	5,4	IGLV10-AAO*01	IGLJ3*01	SAWDTSLSAWV	11	95,6	4,4
RM54C	6454	VL	Unknown	LJI.Rh_IGHV5.157_S5960	IGHD2-30*01,IGHD2-I5	IGHJ4*01	AVPDCSDSGCSSHFDD	16	93,8	6,2	IGLV1-ACR*01,LJI.Rh_IGLV1.69	IGLJ6-S6	SAWDSSLNFHV	11	95,3	4,7
RM35A1	43335	VL	V1 region	IGHV3-ABA*01,IGHV3-Korf20	IGHD3-21*01,LJI.Rh_IGHD3.17	IGHJ4*01	TRTWGDYFAD	10	93,5	6,5	IGLV1-S16	IGLJ3*01	ATWDDALSGRV	11	95,3	4,7
RM35A2	43335	VL	V1 region	IGHV3-ABA*01,IGHV3-Korf20	IGHD3-21*01,LJI.Rh_IGHD3.17	IGHJ4*01	TRTLGDYYVD	10	94,9	5,1	IGLV1-S16	IGLJ3*01	AAWDDNVSGRV	11	95,9	4,1
RM35B1	43335	VL	289	IGHV4-AFU*01	IGHD4-27*02,IGHD6-38*01	IGHJ4*01	ARPTGSSYLFPYYFDS	16	90,9	9,1	IGLV1-S16	IGLJ1-S1	AAWDDSLRGYI	11	97,6	2,4
RM35B2	43335	VL	289	IGHV4-AFU*01	LJI.Rh_IGHD1.8	IGHJ4*01	TRPTGSSWLAPYYFDS	16	91,9	8,1	IGLV1-S16	IGLJ1-S1	ASWDDSLNNYI	11	95,2	4,8
RM35B3	43335	VL	289	IGHV4-AFU*01	IGHD6-11*01	IGHJ4*01	TRPTGSSWLNPYYFDF	16	92,3	7,7	IGLV1-S16	IGLJ1-S1	AAWDDRLGAYI	11	97,9	2,1
RM35C	43335	VL	289	IGHV4-AFU*01	IGHD6-11*01	IGHJ4*01	ARPTGSSWLSPYYFDF	16	96,6	3,4	IGLV1-S16	IGLJ1-S1	AAWDDSLNGYI	11	98,3	1,7
RM46A1	6446	VK	289	LJI.Rh_IGHV4.79.a_S9501	n/a	IGHJ5-2*01	ARKDDSLDV	9	89,1	10,9	LJI.Rh_IGKV1.50	IGKJ2-I2	QQYNSDPYN	9	95,1	4,9
RM46A2	6446	VK	289	LJI.Rh_IGHV4.79.a_S9501	n/a	IGHJ5-2*01	ARKDESLDV	9	92,9	7,1	LJI.Rh_IGKV1.50	IGKJ2-I2	QQYDSDPYN	9	92,6	7,4
RM46A3	6446	VK	289	LJI.Rh_IGHV4.79.a_S9501	IGHD3-9*01,LJI.Rh_IGHD3.15	IGHJ5-2*01	ARKDESFDV	9	92,5	7,5	LJI.Rh_IGKV1.50	IGKJ2-I2	QQYDSDSYS	9	93,6	6,4
RM46A4	6446	VK	289	LJI.Rh_IGHV4.79.a_S9501	n/a	IGHJ5-2*01	ARKDDSLDV	9	89,1	10,9	LJI.Rh_IGKV1.50	IGKJ2-I2	QRYNGDPYD	9	93,7	6,3
RM46A5	6446	VK	289	LJI.Rh_IGHV4.79.a_S9501	IGHD3-9*01,LJI.Rh_IGHD3.15	IGHJ5-2*01	ARKEETLDV	9	89,1	10,9	LJI.Rh_IGKV1.50	IGKJ2-I2	QQYDSDPYN	9	95,1	4,9
RM46A6	6446	VK	289	LJI.Rh_IGHV4.79.a_S9501	n/a	IGHJ5-2*01	AKKEDSFDV	9	92,5	7,5	LJI.Rh_IGKV1.50	IGKJ2-I2	QQYDSDSFT	9	94,3	5,7
RM46A7	6446	VK	289	LJI.Rh_IGHV4.79.a_S9501	n/a	IGHJ5-2*01	ARKDDSLDV	9	88,8	11,2	LJI.Rh_IGKV1.50	IGKJ2-I2	QQYNSDPYN	9	94,4	5,6
RM46A18	6446	VK	289	LJI.Rh_IGHV4.79.a_S9501	IGHD3-36*01,IGHD3-I4	IGHJ5-2*01	AKLEVSLDV	9	92,8	7,2	LJI.Rh_IGKV1.50	IGKJ2-I2	QQYNSDPYS	9	95,1	4,9
RM46A9	6446	VK	289	LJI.Rh_IGHV4.79.a_S9501	IGHD3-9*01,LJI.Rh_IGHD3.15	IGHJ5-2*01	ARKEETLDV	9	89,1	10,9	LJI.Rh_IGKV1.50	IGKJ2-I2	QQYDSDPYN	9	95,1	4,9
RM46A10	6446	VK	289	LJI.Rh_IGHV4.79.a_S9501	IGHD4-27*02,IGHD6-11*01,IGHD6-29*01	IGHJ5-2*01	ARKQDSLDV	9	91,5	8,5	IGKV1-AAY*01	IGKJ2-I2	QQYNSDPYN	9	93,7	6,3
RM46A11	6446	VK	289	LJI.Rh_IGHV4.79.a_S9501	n/a	IGHJ5-2*01	AKKEDSLDV	9	92,1	7,9	IGKV1-AAL*01_S2543	IGKJ2-I2	QQYNSDPYR	9	93,7	6,3
RM46B1	6446	VL	289	LJI.Rh_IGHV5.157_S5960	IGHD2-30*01,IGHD2-I5	IGHJ4*01	AIPHCSDTDCPSHFDY	16	95,9	4,1	IGLV1-ACR*01	IGLJ2-S2	SSWDNSLSTL	10	96,3	3,7
RM46B2	6446	VL	289	LJI.Rh_IGHV5.157_S5960	IGHD2-30*01,IGHD2-I5	IGHJ4*01	AIPHCSDTDCPSRFDY	16	94,1	5,9	IGLV1-ACR*01	IGLJ2-S2	SSWDNSLSTL	10	96,6	3,4
RM46B3	6446	VL	289	LJI.Rh_IGHV5.157_S5960	IGHD2-30*01,IGHD2-I5	IGHJ4*01	AIPHCSDTDCPSHFDY	16	95,9	4,1	IGLV1-ACR*01	IGLJ2-S2	SSWDNSLSTL	10	97,3	2,7
