# Supplementary material for: Antibody responses induced by SHIV infection are more focused than those induced by soluble native HIV-1 envelope trimers in non-human primates
Source: PLoS Pathog. 2021 Aug 25;17(8):e1009736. doi: 10.1371/journal.ppat.1009736 (PMC8423243; doi:10.1371/journal.ppat.1009736)
Supplement: S2 Table — (PDF) [file ppat.1009736.s006.pdf]

Immunization

Animal ID	Antibody ID	Epitope	BG505 SOSIP v5.2	N611Q	S241N	P291S	S241N + P291S	T465N	133aN	gp120	gp120 7C3
ROp15	RM15A	Base	1,0	0,82	0,88	0,92	0,79	0,93	0,89	0,13	0,13
	RM15B	Base	1,0	0,37	0,77	0,76	0,83	0,75	ND	0,10	0,11
	RM15C	V1 region	1,0	0,63	0,84	0,89	0,85	0,84	1,1	0,082	0,079
	RM15D	Base	1,0	0,84	0,92	0,94	0,91	0,92	0,94	0,083	0,077
	RM15E	C3/V5	1,0	0,70	0,74	0,73	0,72	0,66	1,1	0,85	0,10
	RM15F	gp41/gp120 interface	1,0	0,89	0,59	0,80	0,58	0,83	0,92	0,50	0,36
	RM15G	Base	1,0	0,55	0,84	0,81	0,79	0,74	ND	0,11	0,10
	RM15H	Base	1,0	0,77	0,95	0,93	0,84	0,78	ND	0,14	0,11
	RM15I	241/289	1,0	0,68	0,55	0,78	0,47	0,80	1,0	0,12	0,12
	RM15J	Base	1,0	0,12	0,89	0,83	0,81	0,69	0,96	0,12	0,11
	RM15K	Unknown	1,0	0,63	0,87	0,82	0,82	0,96	1,2	0,13	0,14
	RM15L	Base	1,0	0,81	1,1	1,1	1,1	1,1	ND	0,19	0,21
	RM15M	Base	1,0	0,78	0,86	0,88	0,95	0,91	ND	0,10	0,10
	RM15N	Base	1,0	0,66	0,90	0,89	0,89	0,95	0,70	0,13	0,12
	RM15O	Base	1,0	1,4	1,1	1,4	1,3	1,1	ND	0,45	0,45
99-12	RM12A	Base	1,0	0,69	0,96	0,94	0,96	0,92	1,1	0,081	0,072
	RM12B	Base	1,0	0,68	0,90	1,0	1,1	0,90	1,6	1,4	1,2
	RM12C	241/289	1,0	0,93	0,88	0,26	0,24	0,89	0,85	0,80	0,47
	RM12D	241/289	1,0	0,83	0,79	0,25	0,26	0,96	0,84	0,82	0,48
	RM12E	241/289	1,0	0,90	0,85	0,26	0,29	0,79	1,1	0,50	0,23
	RM12F	N611/FP	1,0	0,91	0,82	0,70	0,69	0,65	1,0	0,11	0,11
	RM12G	Base	1,0	0,72	0,94	0,84	0,84	0,75	ND	0,072	0,057
	RM12H	Base	1,0	0,87	0,91	0,88	0,86	0,86	1,1	0,092	0,10
	RM12I	Unknown	1,0	0,71	0,88	1,1	1,3	0,89	1,05	1,8	1,6
	RM12J	Base	1,0	0,35	0,95	0,97	0,94	0,91	0,92	0,070	0,055
	RM12K	gp41/gp120 interface	1,0	0,80	0,87	0,87	0,86	0,88	0,88	0,10	0,086
	RM12L	Base	1,0	0,59	0,84	0,88	0,84	0,87	ND	0,10	0,12
	RM12M	Base	1,0	0,78	0,96	0,96	1,1	1,2	ND	0,22	0,31
	RM12N	Base	1,0	0,73	0,99	1,2	1,2	1,02	ND	0,36	0,40
	RM12O	Base	1,0	0,91	0,94	0,97	0,89	0,95	ND	0,11	0,10
	RM12P	Base	1,0	0,86	1,1	1,1	0,96	1,02	ND	0,15	0,22
	RM12Q	Base	1,0	0,76	0,84	0,89	0,84	0,82	ND	0,11	0,12

Fold difference in Area Under the Curve	
	≤0.33
	>0.33 ≤0.60
	>0.60 ≤1.0
	>1.0
	No Data (ND)

Infection

Animal ID	Antibody ID	Epitope	BG505 SOSIP v5.2	N611Q	S241N	P291S	S241N + P291S	T465N	133aN	gp120	gp120 7C3
6454	RM54A	241/289	1,0	0,78	0,77	0,30	0,25	0,86	1,1	0,38	0,35
	RM54B1	V1 region	1,0	0,80	0,78	1,0	1,1	0,61	0,66	0,20	0,15
	RM54B2	V1 region	1,0	0,99	1,0	1,0	1,1	0,80	0,70	0,36	0,27
	RM54B3	V1 region	1,0	0,88	1,1	0,79	0,92	0,85	0,28	0,23	0,27
	RM54B4	V1 region	1,0	0,94	1,0	1,2	1,1	1,4	0,20	0,18	0,12
	RM54B5	V1 region	1,0	1,1	1,1	1,1	1,0	0,73	0,26	0,26	0,35
	RM54B6	V1 region	1,0	1,1	1,0	1,0	1,1	0,85	0,10	0,40	0,30
	RM54B7	V1 region	1,0	0,93	1,0	0,95	0,92	0,86	0,13	0,47	0,29
	RM54B8	V1 region	1,0	0,76	0,76	1,0	1,1	0,87	0,15	0,29	0,21
	RM54B9	V1 region	1,0	0,88	0,83	0,90	1,0	0,85	0,14	0,16	0,16
	RM54B10	V1 region	1,0	1,1	1,2	1,5	1,5	1,0	0,12	0,19	0,22
	RM54B11	V1 region	1,0	1,0	1,0	1,0	1,1	0,94	0,12	0,38	0,27
	RM54B12	V1 region	1,0	0,74	1,2	1,0	1,2	0,83	0,14	0,19	0,19
	RM54B13	V1 region	1,0	0,88	1,0	0,93	1,1	0,65	0,46	0,17	0,17
	RM54B14	V1 region	1,0	1,1	1,1	1,1	1,1	1,0	0,05	0,24	0,29
	RM54B15	V1 region	1,0	1,0	0,91	1,0	1,2	0,52	0,54	0,25	0,52
	RM54B16	V1 region	1,0	1,0	1,1	1,1	1,1	0,91	0,55	0,40	0,30
	RM54B17	V1 region	1,0	1,3	1,1	1,3	1,6	1,2	0,42	0,32	0,39
	RM54B18	V1 region	1,0	1,0	1,0	1,0	1,1	0,84	0,42	0,20	0,22
	RM54B19	V1 region	1,0	0,93	0,83	1,0	1,0	0,89	0,20	0,31	0,31
	RM54B20	V1 region	1,0	0,88	1,1	1,0	1,1	0,88	0,39	0,22	0,19
	RM54B21	V1 region	1,0	0,88	1,0	0,93	1,0	0,88	0,59	0,43	0,36
	RM54B22	V1 region	1,0	0,85	1,0	0,88	0,95	0,80	0,62	0,27	0,26
	RM54B23	V1 region	1,0	0,93	0,93	0,92	1,0	0,93	0,41	0,46	0,42
	RM54C	Unknown	1,0	3,2	1,8	2,0	2,6	2,0	1,2	4,6	3,4
43335	RM35A1	V1 region	1,0	0,67	0,65	0,78	0,80	0,64	0,25	0,16	0,13
	RM35A2	V1 region	1,0	0,98	0,86	1,0	1,0	0,95	ND	0,35	0,33
	RM35B1	241/289	1,0	0,69	0,47	0,10	0,091	0,77	0,92	0,32	0,38
	RM35B2	241/289	1,0	0,60	0,17	0,094	0,065	0,65	0,93	0,28	0,32
	RM35B3	241/289	1,0	0,65	0,24	0,13	0,10	0,72	1,0	0,37	0,42
	RM35C	241/289	1,0	0,90	0,38	0,36	ND	1,0	1,0	0,37	0,42
6446	RM46A1	241/289	1,0	0,83	0,83	0,33	0,33	0,88	1,2	0,81	0,81
	RM46A2	241/289	1,0	0,78	0,68	0,38	0,37	0,78	0,93	0,58	0,62
	RM46A3	241/289	1,0	0,73	0,82	0,27	0,27	0,84	1,0	0,72	0,58
	RM46A4	241/289	1,0	0,71	0,66	0,25	0,23	0,72	0,89	0,69	0,72
	RM46A5	241/289	1,0	0,63	0,64	0,087	0,079	0,70	0,85	0,63	0,59
	RM46A6	241/289	1,0	0,77	0,58	0,11	0,13	0,76	0,85	0,60	0,61
	RM46A7	241/289	1,0	0,91	0,84	0,38	0,34	0,95	0,92	0,73	0,79
	RM46A8	241/289	1,0	0,71	0,48	0,16	0,20	0,71	1,1	0,29	0,38
	RM46A9	241/289	1,0	0,66	0,65	0,090	0,088	0,92	1,1	0,56	0,54
	RM46A10	241/289	1,0	0,65	0,63	0,21	0,26	0,69	0,92	0,60	0,55
	RM46A11	241/289	1,0	1,0	0,87	0,18	0,26	0,86	0,87	0,79	0,75
	RM46B1	241/289	1,0	1,4	1,0	1,1	1,6	1,4	1,2	2,2	1,6
	RM46B2	241/289	1,0	1,1	1,0	0,88	0,95	1,1	1,1	1,7	1,1
	RM46B3	241/289	1,0	1,9	1,5	1,6	1,9	1,3	1,0	3,8	2,5
