# Supplementary material for: Antibody responses induced by SHIV infection are more focused than those induced by soluble native HIV-1 envelope trimers in non-human primates
Source: PLoS Pathog. 2021 Aug 25;17(8):e1009736. doi: 10.1371/journal.ppat.1009736 (PMC8423243; doi:10.1371/journal.ppat.1009736)
Supplement: S3 Table — (PDF) [file ppat.1009736.s007.pdf]

	Primer	Sequence 5' to 3'
**Heavy chain 1st PCR primers**	5'VH1.L1	ATGGACTKGACCTGGAGG
**Forward primers**	5'VH2.L1	ATGGACACGCTTTGCTCC
	5'VH3A.L1	ATGGAGTTKGGGCTGAGCTG
	5'VH3B.L1	ATGGAGTTTGKRCTGAGCTGG
	5'VH3C.L1	ATGGAGTCRTGGCTGAGCTGG
	5'VH3D.L1	ATGGAGTTTGTGCTGAGTTTGG
	5'VH4.L1	ATGAAGCACCTGTGGTTC
	5'VH5A.L1	ATGGGGTCAACTGCCATC
	5'VH5B.L1	ATGGGGTCCACCGTCACC
	5'VH6.L1	ATGTCTGTCTCCTTCCTCA
	5'VH7.L1	ATGGACCTCACCTGGAGC
**Reverse primer**	3'IgG(Outer)	GGAAGGTGTGCACGCCGCTGGTC

	Primer	Sequence 5' to 3'
**Heavy chain 2nd PCR primers**	5'VH1A.SE	TGGCAGCAGCTACAGGTGC
**Forward primers**	5'VH1B.SE	TGACAGCAGCTACAGGCGC
	5'VH1C.SE	TGGCAGCAGCAACAGGCAC
	5'VH2.SE	GTCCCGTCCTGGGTCTTGTC
	5'VH3A.SE	GCTGTTTGGAGAGGTGTCCAGTGTG
	5'VH3B.SE	GCCATATTAGAAGGTGTCCAGTGTG
	5'VH3C.SE	GCTCTTTTGAAAGGTGTCCAGTGTG
	5'VH3D.SE	GCTATTTTAAGAGGTGTCCAGTGTG
	5'VH3E.SE	GCTATTTTAAAAGGTGTCCAGTGTG
	5'VH4.SE	AGCTCCCAGATGGGTCYTGTCC
	5'VH5.SE	GCTGTTCTCCARGGAGTCTGTG
	5'VH6.SE	GGCCTCCCATGGGGTGTC
	5'VH7A.SE	GCAGCAACAGGTGCCCACTC
	5'VH7B.SE	GCAGCAACAGGCACCCACTC
**Reverse primers**	3'IgG(Inner)	GTTCAGGGAAGTAGTCCTTGAC

	Primer	Sequence 5' to 3'
**Heavy chain 3th PCR primers**	5'AgeI.VH1A	CATCCTTTTTCTAGTAGCAACTGCAACCGGTGTACACTCGCAGGTGCAGCTGGTGCAGTC
**Forward primers**	5'AgeI.VH1B	CATCCTTTTTCTAGTAGCAACTGCAACCGGTGTACACTCGCAGGTCCAGCTGGTGCAGTC
	5'AgeI.VH1C	CATCCTTTTTCTAGTAGCAACTGCAACCGGTGTACACTCGGAGGTCCAGCTGGTGCAGTC
	5'AgeI.VH2A	CATCCTTTTTCTAGTAGCAACTGCAACCGGTGTACACTCGCAGGTGACCTTGAAGGAGTC
	5'AgeI.VH2B	CATCCTTTTTCTAGTAGCAACTGCAACCGGTGTACACTCGCAGGTCACCTTGAAGGAGTC
	5'AgeI.VH3A	CATCCTTTTTCTAGTAGCAACTGCAACCGGTGTACACTCGGAGGTGCAGCTGGTGGAGTC
	5'AgeI.VH3B	CATCCTTTTTCTAGTAGCAACTGCAACCGGTGTACACTCGGAGGTGCGGCTGGTGGAGTC
	5'AgeI.VH3C	CATCCTTTTTCTAGTAGCAACTGCAACCGGTGTACACTCGGAGGTGCAGCTGGTGGCGTA
	5'AgeI.VH3D	CATCCTTTTTCTAGTAGCAACTGCAACCGGTGTACACTCGGAGGCGCAGCTGATGGAAAC
	5'AgeI.VH3E	CATCCTTTTTCTAGTAGCAACTGCAACCGGTGTACACTCGGAGGTGCAGCTGGCGGAGTC
	5'AgeI.VH3F	CATCCTTTTTCTAGTAGCAACTGCAACCGGTGTACACTCGGAGGTGCAGCTAGTGGAGTC
	5'AgeI.VH3G	CATCCTTTTTCTAGTAGCAACTGCAACCGGTGTACACTCGGACGTGCAGCTGGTGGAGTC
	5'AgeI.VH3H	CATCCTTTTTCTAGTAGCAACTGCAACCGGTGTACACTCGGAGGTGCAGTTGGTGGAGTC
	5'AgeI.VH3I	CATCCTTTTTCTAGTAGCAACTGCAACCGGTGTACACTCGGTGGAGCAGCTGGTGGAGTC
	5'AgeI.VH3J	CATCCTTTTTCTAGTAGCAACTGCAACCGGTGTACACTCGGAGGTGCAGCTGGTAGAGTC
	5'AgeI.VH3K	CATCCTTTTTCTAGTAGCAACTGCAACCGGTGTACACTCGGAAGTGCAGTTGGTGGAGTC
	5'AgeI.VH3L	CATCCTTTTTCTAGTAGCAACTGCAACCGGTGTACACTCGGAGGTGCAGCGGGTGGAGTC
	5'AgeI.VH3M	CATCCTTTTTCTAGTAGCAACTGCAACCGGTGTACACTCGGAGGTGCAACTGGTGGAGTC
	5'AgeI.VH4A	CATCCTTTTTCTAGTAGCAACTGCAACCGGTGTACACTCGCAGCTGCAGCTGCAGGAGTC
	5'AgeI.VH4B	CATCCTTTTTCTAGTAGCAACTGCAACCGGTGTACACTCGCAGGTGCAGCTGCAGGAGTC
	5'AgeI.VH5A	CATCCTTTTTCTAGTAGCAACTGCAACCGGTGTACACTCGGAGGTGCAGCTGGTGCAGTC
	5'AgeI.VH6A	CATCCTTTTTCTAGTAGCAACTGCAACCGGTGTACACTCGCAGGTGCAGCTGCAGGAGTC
**Reverse primers**	3'SalI.JH1/4/5	GGAAGACCGATGGGCCCTTGGTCGACGCTGAGGAGACGGTGACCAG
	3'SalI.JH2	GGAAGACCGATGGGCCCTTGGTCGACGCTGAGGAGATGGTGATTGGG
	3'SalI.JH3	GGAAGACCGATGGGCCCTTGGTCGACGCTGAAGAGACGGTGACCCTG
	3'SalI.JH6	GGAAGACCGATGGGCCCTTGGTCGACGCTGAGGAGACGGTGACGACG

	Primer	Sequence 5' to 3'
**Heavy chain 1st PCR primers**	5'VH1.L1	ATGGACTKGACCTGGAGG
**Forward primers**	5'VH2.L1	ATGGACACGCTTTGCTCC
	5'VH3A.L1	ATGGAGTTKGGGCTGAGCTG
	5'VH3B.L1	ATGGAGTTTGKRCTGAGCTGG
	5'VH3C.L1	ATGGAGTCRTGGCTGAGCTGG
	5'VH3D.L1	ATGGAGTTTGTGCTGAGTTTGG
	5'VH4.L1	ATGAAGCACCTGTGGTTC
	5'VH5A.L1	ATGGGGTCAACTGCCATC
	5'VH5B.L1	ATGGGGTCCACCGTCACC
	5'VH6.L1	ATGTCTGTCTCCTTCCTCA
	5'VH7.L1	ATGGACCTCACCTGGAGC
**Reverse primer**	3'IgG(Outer)	GGAAGGTGTGCACGCCGCTGGTC

	Primer	Sequence 5' to 3'
**Heavy chain 2nd PCR primers**	5'VH1A.SE	TGGCAGCAGCTACAGGTGC
**Forward primers**	5'VH1B.SE	TGACAGCAGCTACAGGCGC
	5'VH1C.SE	TGGCAGCAGCAACAGGCAC
	5'VH2.SE	GTCCCGTCCTGGGTCTTGTC
	5'VH3A.SE	GCTGTTTGGAGAGGTGTCCAGTGTG
	5'VH3B.SE	GCCATATTAGAAGGTGTCCAGTGTG
	5'VH3C.SE	GCTCTTTTGAAAGGTGTCCAGTGTG
	5'VH3D.SE	GCTATTTTAAGAGGTGTCCAGTGTG
	5'VH3E.SE	GCTATTTTAAAAGGTGTCCAGTGTG
	5'VH4.SE	AGCTCCCAGATGGGTCYTGTCC
	5'VH5.SE	GCTGTTCTCCARGGAGTCTGTG
	5'VH6.SE	GGCCTCCCATGGGGTGTC
	5'VH7A.SE	GCAGCAACAGGTGCCCACTC
	5'VH7B.SE	GCAGCAACAGGCACCCACTC
**Reverse primers**	3'IgG(Inner)	GTTCAGGGAAGTAGTCCTTGAC

	Primer	Sequence 5' to 3'
**Heavy chain 3th PCR primers**	5'AgeI.VH1A	CATCCTTTTTCTAGTAGCAACTGCAACCGGTGTACACTCGCAGGTGCAGCTGGTGCAGTC
**Forward primers**	5'AgeI.VH1B	CATCCTTTTTCTAGTAGCAACTGCAACCGGTGTACACTCGCAGGTCCAGCTGGTGCAGTC
	5'AgeI.VH1C	CATCCTTTTTCTAGTAGCAACTGCAACCGGTGTACACTCGGAGGTCCAGCTGGTGCAGTC
	5'AgeI.VH2A	CATCCTTTTTCTAGTAGCAACTGCAACCGGTGTACACTCGCAGGTGACCTTGAAGGAGTC
	5'AgeI.VH2B	CATCCTTTTTCTAGTAGCAACTGCAACCGGTGTACACTCGCAGGTCACCTTGAAGGAGTC
	5'AgeI.VH3A	CATCCTTTTTCTAGTAGCAACTGCAACCGGTGTACACTCGGAGGTGCAGCTGGTGGAGTC
	5'AgeI.VH3B	CATCCTTTTTCTAGTAGCAACTGCAACCGGTGTACACTCGGAGGTGCGGCTGGTGGAGTC
	5'AgeI.VH3C	CATCCTTTTTCTAGTAGCAACTGCAACCGGTGTACACTCGGAGGTGCAGCTGGTGGCGTA
	5'AgeI.VH3D	CATCCTTTTTCTAGTAGCAACTGCAACCGGTGTACACTCGGAGGCGCAGCTGATGGAAAC
	5'AgeI.VH3E	CATCCTTTTTCTAGTAGCAACTGCAACCGGTGTACACTCGGAGGTGCAGCTGGCGGAGTC
	5'AgeI.VH3F	CATCCTTTTTCTAGTAGCAACTGCAACCGGTGTACACTCGGAGGTGCAGCTAGTGGAGTC
	5'AgeI.VH3G	CATCCTTTTTCTAGTAGCAACTGCAACCGGTGTACACTCGGACGTGCAGCTGGTGGAGTC
	5'AgeI.VH3H	CATCCTTTTTCTAGTAGCAACTGCAACCGGTGTACACTCGGAGGTGCAGTTGGTGGAGTC
	5'AgeI.VH3I	CATCCTTTTTCTAGTAGCAACTGCAACCGGTGTACACTCGGTGGAGCAGCTGGTGGAGTC
	5'AgeI.VH3J	CATCCTTTTTCTAGTAGCAACTGCAACCGGTGTACACTCGGAGGTGCAGCTGGTAGAGTC
	5'AgeI.VH3K	CATCCTTTTTCTAGTAGCAACTGCAACCGGTGTACACTCGGAAGTGCAGTTGGTGGAGTC
	5'AgeI.VH3L	CATCCTTTTTCTAGTAGCAACTGCAACCGGTGTACACTCGGAGGTGCAGCGGGTGGAGTC
	5'AgeI.VH3M	CATCCTTTTTCTAGTAGCAACTGCAACCGGTGTACACTCGGAGGTGCAACTGGTGGAGTC
	5'AgeI.VH4A	CATCCTTTTTCTAGTAGCAACTGCAACCGGTGTACACTCGCAGCTGCAGCTGCAGGAGTC
	5'AgeI.VH4B	CATCCTTTTTCTAGTAGCAACTGCAACCGGTGTACACTCGCAGGTGCAGCTGCAGGAGTC
	5'AgeI.VH5A	CATCCTTTTTCTAGTAGCAACTGCAACCGGTGTACACTCGGAGGTGCAGCTGGTGCAGTC
	5'AgeI.VH6A	CATCCTTTTTCTAGTAGCAACTGCAACCGGTGTACACTCGCAGGTGCAGCTGCAGGAGTC
**Reverse primers**	3'SalI.JH1/4/5	GGAAGACCGATGGGCCCTTGGTCGACGCTGAGGAGACGGTGACCAG
	3'SalI.JH2	GGAAGACCGATGGGCCCTTGGTCGACGCTGAGGAGATGGTGATTGGG
	3'SalI.JH3	GGAAGACCGATGGGCCCTTGGTCGACGCTGAAGAGACGGTGACCCTG
	3'SalI.JH6	GGAAGACCGATGGGCCCTTGGTCGACGCTGAGGAGACGGTGACGACG

	Primer	Sequence 5' to 3'
**Lambda chain 1st PCR primers**	5'VL1.L1	ATGGCCTGGTYYCCTCTC
**Forward primers**	5'VL2/7/10.L1	ATGGCCTGGRCTCTGCTCC
	5'VL3A.L1	ATGGCCTGGATTCCTCTC
	5'VL3B.L1	ATGGCCTGGACCTTTCTC
	5'VL3C.L1	ATGGCCTGGACCCCTCCC
	5'VL4A.L1	ATGGCCTGGGTCTCCTTC
	5'VL4B.L1	ATGGCCTGGACCCCACTC
	5'VL5/11.L1	ATGGCCTGGACTCCTCTC
	5'VL6.L1	ATGGCCTGGGCTCCACTCC
	5'VL8.L1	ATGGCCTGGATGATGCTTC
	5'VL9.L1	ATGGCCTGGGCTCCTCTG
**Reverse primers**	3'Lambda(Outer)	TGTTGCTCTGTTTGGAGGG

	Primer	Sequence 5' to 3'
**Lambda chain 2nd PCR primers**	5'VL1A.SE	CTGTGCAGGGTCCTGGGCC
**Forward primers**	5'VL1B.SE	CTGCACAGGGTCCYGGGCC
	5'VL2.SE	TCACTCAGGGCACAGGATCC
	5'VL3A.SE	CGCCCTCTGCACAGTCTCTGTGG
	5'VL3B.SE	CACTCTCTGCACAGGTTCCGTGG
	5'VL4A.SE	TTCATTTTCTCCACAGGTCTCTGTG
	5'VL4B.SE	CTTCACTGCAGAGGTGTCTCTC
	5'VL5.SE	CACTGCACAGGTTCCCTCTC
	5'VL6.SE	CTGCACAGGGTCTTGGGCTG
	5'VL8.SE	GCTTATGGCTCAGGAGTGGA
**Reverse primers**	3'Lambda(Inner)	AGACACACTAGTGTGGCCTTG

	Primer	Sequence 5' to 3'
**Lambda chain 3th PCR primers**	5'AgeI.VL1A	CATCCTTTTTCTAGTAGCAACTGCAACCGGTGTACACCAGTCTGTGCTGACACA
**Forward primers**	5'AgeI.VL1B	CATCCTTTTTCTAGTAGCAACTGCAACCGGTGTACACCAGTCTGTGCTGACGCA
	5'AgeI.VL1C	CATCCTTTTTCTAGTAGCAACTGCAACCGGTGTACACCAGTCTGTGCTGACTCA
	5'AgeI.VL2A	CATCCTTTTTCTAGTAGCAACTGCAACCGGTGTACACCAGGCTGCCCTGACTCAG
	5'AgeI.VL2B	CATCCTTTTTCTAGTAGCAACTGCAACCGGTGTACACCAGTCTGCCCTGACTCAG
	5'AgeI.VL2C	CATCCTTTTTCTAGTAGCAACTGCAACCGGTGTACACCAGGCTGCCCCGACTCAG
	5'AgeI.VL3A	CATCCTTTTTCTAGTAGCAACTGCAACCGGTGTACACTCCTATGAGCTGACTCAG
	5'AgeI.VL3B	CATCCTTTTTCTAGTAGCAACTGCAACCGGTGTACACTCTTCTGAGCTGACTCAG
	5'AgeI.VL3C	CATCCTTTTTCTAGTAGCAACTGCAACCGGTGTACACTCCTCTGAGCTGACTCAG
	5'AgeI.VL3D	CATCCTTTTTCTAGTAGCAACTGCAACCGGTGTACACTCCTCCGGGCTGACTCAG
	5'AgeI.VL3E	CATCCTTTTTCTAGTAGCAACTGCAACCGGTGTACACTCCTATGAGCTGACACAG
	5'AgeI.VL3F	CATCCTTTTTCTAGTAGCAACTGCAACCGGTGTACACTCTTGTGAACTGACACAG
	5'AgeI.VL3G	CATCCTTTTTCTAGTAGCAACTGCAACCGGTGTACACTCCTATGATGTGACTCAG
	5'AgeI.VL3H	CATCCTTTTTCTAGTAGCAACTGCAACCGGTGTACACTCCTATGATCTGACTCAG
	5'AgeI.VL4A	CATCCTTTTTCTAGTAGCAACTGCAACCGGTGTACACCTGCCTGTGCTGACTCAG
	5'AgeI.VL4B	CATCCTTTTTCTAGTAGCAACTGCAACCGGTGTACACCAGCCTGTGCTGACTCAG
	5'AgeI.VL5A	CATCCTTTTTCTAGTAGCAACTGCAACCGGTGTACACAAGCCTATGCTGACTCAGCC
	5'AgeI.VL5B	CATCCTTTTTCTAGTAGCAACTGCAACCGGTGTACACCAGCCTGTGCTGACTCAGCC
	5'AgeI.VL6A	CATCCTTTTTCTAGTAGCAACTGCAACCGGTGTACACCCCCATTCTGTGTCGGGGTC
	5'AgeI.VL7A	CATCCTTTTTCTAGTAGCAACTGCAACCGGTGTACACCAGGCTGTAGTGACTCAGGA
	5'AgeI.VL8A	CATCCTTTTTCTAGTAGCAACTGCAACCGGTGTACACGAGACTGTGGTGACCCAGGA
	5'AgeI.VL9/11A	CATCCTTTTTCTAGTAGCAACTGCAACCGGTGTACACCAGCCTGTGCTGACTCAGC
	5'AgeI.VL10A	CATCCTTTTTCTAGTAGCAACTGCAACCGGTGTACACCAGGCAGGGCTGACTCAGCC
**Reverse primers**	3'XhoI.CL1	GTTGGCTTGAAGCTCCTCACTCGAGGGCGGGAACAGAGTG
	3'XhoI.CL2/3	GTTGGCTTGAAGCTCCTCACTCGAGGGTGGGAACAGAGTG
	3'XhoI.CL5	GTTGGCTTGAAGCTCCTCACTCGAGGGCGGGAAGAGAGTG
	3'XhoI.CL7	GTTGGCTTGAAGCTCCTCACTCGAGGGCGGAAAGAGAGTG
