# Supplementary material for: Antibody responses induced by SHIV infection are more focused than those induced by soluble native HIV-1 envelope trimers in non-human primates
Source: PLoS Pathog. 2021 Aug 25;17(8):e1009736. doi: 10.1371/journal.ppat.1009736 (PMC8423243; doi:10.1371/journal.ppat.1009736)
Supplement: Attachment — Submitted filename: Response_to_reviewers_Jelle_van_Schooten_et_al_2021.docx [file ppat.1009736.s008.docx]

We appreciate the time and effort the reviewers spent to review our manuscript. We are thankful for the constructive feedback, which will improve the quality of the manuscript. We tried to address each of the reviewers comments. The reviewers text is shown in italic black, our replies are in blue.

**Part I - Summary**
*Reviewer #1: The manuscript by van Gils and colleagues compares side by side the antibody responses generated by BG505 SOSIP trimer immunization and BG505 SHIV infection in NHPs. The authors isolated mAbs from both animal populations and characterized their binding and neutralization profiles in detail. BG505 SHIV infection leads to a more pronounced clonal expansion and, interestingly, to a much lower extend of antibody diversity. BG505 SHIV infection produces mostly neutralizing antibodies targeting the 241/289 glycan hole and the V1 region. In contrast, BG505 SOSIP immunization generated antibodies that target more diverse sites including the glycan hole at N611, C3/V5, the gp41/gp120 interface and the V1 region. Notably, the V1 antibodies were the most potent neutralizing antibodies in both groups. However, neither V1 or other Abs neutralized heterologous viruses as reported in previous approaches and vaccine regimens. A substantial amount of non-neutralizing antibodies was also elicited targeting the base of the soluble SOSIP trimers as reported in previous studies using SOSIP-based soluble trimers.

Overall, the study design is novel and indicates some differences in antibody generation by soluble trimers compared to a natural infection setting within comparable time periods. The data is well analyzed and provides new clues to pursue further immunogen optimization such as diverging the immune response away from the variable V1 epitope towards more conserved known neutralizing Ab epitopes.*

*Reviewer #2: This paper entitled “Antibody responses induced by SHIV infection are more focused than those induced by soluble native HIV-1 envelope trimers in non-human primates” aims at comparing vaccine-induced antibody responses to BG505 SOSIP.664 immunogen to those induced by natural infection by a BG505 SHIV. They report that BG505 SHIV infection lead to more clonal expansion but less antibody diversity compared to the BG505 SOSIP immunization and that the SHIV elicited Mabs target less diverse epitopes. None are broad neutralizing antibodies, unfortunately.

This is a well written paper with a straightforward story.*

We thank the reviewers for the accurate summary, kind words, and emphasizing the implications of this study for the field.

***Part II – Major Issues: Key Experiments Required for Acceptance*** *Reviewer #1: The BG505 SHIV probably does not contain any SOSIP modifications. The authors should explain whether the differences in antibody responses observed maybe due to the SOSIP stabilization modifications. Indeed the stabilized version may just present “better” defined epitopes and hence a broader Ab response.*

This is a valid argument and we have now included an extra paragraph regarding this point (lines 399-411). We have also clarified in the text that by using SOSIP trimers as a sorting probe we have filtered out antibodies against aberrant forms of Env (lines 172-174).

*Reviewer #2: However it is unclear what the advance is here and how useful it will be to advance HIV vaccine design.*
We have added some more discussion points on how the data can help improve HIV vaccines throughout the article (lines 419-420 and 455-456). In our discussion we make several arguments how our data can contribute to future HIV vaccine design, such as base masking and glycan modifications which is summarized in the last paragraph of our discussion (lines 492-494).

*Can the authors explain why they used different sorting strategy - does this not bias the result?*

We regret that this was not clear from the text. We now explain more clearly that the mutant used to sort the PBMCs from infected animals does not select for additional B cells, but only provides information on the proportion of B cells targeting the strain-specific 241 glycan hole epitope. This mutant was not available yet at the time when the PBMCs of the immunized animals were sorted (lines 176-178).
 *the authors could explain why they think there are differences in the Nab elicited - for some it is obvious but what about V1 vs 611 - maybe less accessible and the C3/V5…?*
This is a very interesting discussion point that we have now incorporated in the discussion (lines 408-411).
 *It could be interesting to understand the basis for the autologous neut (and not breadth) - higher resolution structure might provide that information.*

We agree that higher resolution data is definitely helpful to pin-point the molecular mechanism of autologous neutralization and could explain the absence of neutralization breadth. However, several papers have already been published that describe higher resolution structures of (N)Abs isolated from BG505 immunized NHPs (Cottrell *et al.* 2020, Nogal *et al.* 2020, Antanasijevic et al. 2021) and rabbits (Nogal *et al.* 2020). Additional structure information on (N)Abs and their interaction with BG505 would be valuable for better interpretation of the findings. We now discuss the results of these structural studies to show why these responses were not broadly neutralizing and how they contribute to understanding of our results (lines 442-443 and 444-446 and 478-480). However, the goal of our manuscript is to compare the antibody response between antibodies induced by immunization and infection and we believe that higher-resolution data is not essential to the message we want to tell in this manuscript and will require significant additional work.

***Part III – Minor Issues: Editorial and Data Presentation Modifications*** *Reviewer #1: Line 528, the SOSP version should be indicated in the methods section.*

We have now included the SOSIP version in the methods (lines 528).

*The authors discuss strategies to focus the immune response. Within this context, the approach described by Dubrovskaya et al 2019 could be discussed as well.*

We agree with the reviewer on this point and have now discussed this approach as well (lines 454-456).

*Reviewer #2: It will be nice to remind the readers of the previous immunizations done and summarize the work that has been previously published (including SHIV infections).*
In the introduction, we have included an elaborate description of previous work on BG505 SOSIP immunization in animal models, especially the work on monoclonal antibody isolation and characterization that were elicited in these animals (lines 101-132). We have added a short summary in the introduction about the polyclonal antibody response in BG505 SHIV infected non-human primates (lines 142-144). To date, no study has been published yet about monoclonal antibody characterization after BG505 SHIV infection, this has been emphasized in the final section of the introduction (lines 144-146).

*The BG505 SHIV model and results have been published elsewhere and it is not surprising that no base directed Mabs will be elicited since it is indeed not present in the SHIV compared to the soluble BG505 SOSIP. The authors could mention this but they seem to be making a case which is likely not needed. It seems quite surprising that reports using BG505 SOSIP with glycans hiding the base of the trimer (for example) to prevent the elicitation of “base-directed Mab” have yet to be published.*

We recognize the comment of the reviewer and this is why we mention that base binding antibodies are not elicited in the SHIV infected NHPs for this reason (lines 345-348). Although this has been shown before, we believe that it was necessary to make this case again to confirm the findings of these studies. Especially because it makes up such a large proportion of the immune response in the BG505 SOSIP immunized non-human primates and not in the BG505 SHIV infected non-human primates.

We have now more elaborately discussed studies hiding the base including glycan-masking of the base as mentioned by the reviewer and agree that such studies should be performed and published (lines 422-423).

*Figure legend - more captions will be nice - animal ID or something.*
We have now adapted the figure legends to contain a better description of the results displayed in the figures (lines 854-905).

*Fig 2A - Please state the numbers of autologous nAb?*

We have included the number of NAbs, and non-NAbs in the figure.

*Although the analysis is interesting, why not show directly the neutralization and what are the properties of the Nab that correlate with neutralization*
The IC_50_ for each of the individual NAbs are shown in figure 2B. In figure 4 we show the neutralization potency in relation to the targeted epitope. We have added CDRH3 length and V-gene usage properties in relationship to neutralization in the text (lines 232-233 and 237-239 and 284-285 and 358-359 and 352-353).

*Between fig 1 and 2, which ones are the autologous N Mab?*

We did not highlight the autologous neutralizing mAbs in figure 1 because we feel that it will not add any value to the figure, because we see no correlation between CDRH3 length. We now mention the CDRH3 lengths of the neutralizing mAbs in the text (lines 233-234 and 238-240). With regards to the V-gene usage and targeted epitopes we see some interesting patterns which we have now also included in the text (lines 285-286 and 355-356).

*Seems that the VH3 paired with Vl10 in 6454, VH3 paired with Vl1 in 43335 and vh5 paired with Vl1 in 6446… Will be interesting to know if they correlated. Assuming all the nab are shown in 2B.*
The reviewer is correct about the observed gene usage, however all the antibodies per animal belong to one single lineage. Therefore, we cannot say anything about a possible correlation between V-gene usage and neutralizing ability. To be able to do this, a larger set of antibodies from different lineages is needed, but unfortunately we were not able to generate more antibodies from these animals. We now mention V-gene usage in relation to their epitopes (see response to previous comment).

*Figure 3B, state the animal ID as well. Color code the Fab vs Env*
We now have stated the animal ID in this figure. We have also color coded the fabs attached to BG505 SOSIP. We have changed the color legend for the different epitopes for figure 3A and 4 so they now match the colors used to designated similar epitopes used in other publications (Nogal *et al.* 2020, Antanasijevic et al. 2021). We believe this will make it easier for the reader to compare the data with data from other manuscripts.

*typo line 394 - longitudinal*

We have corrected this typo (line 394).
